# Predicting endometrial cancer subtypes and molecular features from histopathology images using multi-resolution deep learning models

**DOI:** 10.1016/j.xcrm.2021.100400

**Published:** 2021-09-23

**Authors:** Runyu Hong, Wenke Liu, Deborah DeLair, Narges Razavian, David Fenyö

**Affiliations:** 1 Institute for Systems Genetics, NYU Grossman School of Medicine, New York, NY 10016, USA; 2 Department of Biochemistry and Molecular Pharmacology, NYU Grossman School of Medicine, New York, NY 10016, USA; 3 Department of Pathology, NYU Grossman School of Medicine, New York, NY 10016, USA; 4 Department of Population Health, NYU Grossman School of Medicine, New York, NY 10016, USA; 5 Department of Radiology, NYU Grossman School of Medicine, New York, NY 10016, USA

**Keywords:** endometrial carcinoma, cancer imaging, deep learning, computational pathology, computational biology, cancer genomics

## Abstract

The determination of endometrial carcinoma histological subtypes, molecular subtypes, and mutation status is critical for the diagnostic process, and directly affects patients’ prognosis and treatment. Sequencing, albeit slower and more expensive, can provide additional information on molecular subtypes and mutations that can be used to better select treatments. Here, we implement a customized multi-resolution deep convolutional neural network, Panoptes, that predicts not only the histological subtypes but also the molecular subtypes and 18 common gene mutations based on digitized H&E-stained pathological images. The model achieves high accuracy and generalizes well on independent datasets. Our results suggest that Panoptes, with further refinement, has the potential for clinical application to help pathologists determine molecular subtypes and mutations of endometrial carcinoma without sequencing.

## Highlights

CNN models predict subtypes and mutations in endometrial cancer based on H&E images
Multi-resolution CNN models perform better on H&E images than single-resolution ones
Feature extraction suggests CNN models use human interpretable tumor features
Tumor grade distinguishes CNV-H molecular subtype from endometrioid histology samples

## Graphical abstract

**Figure:**
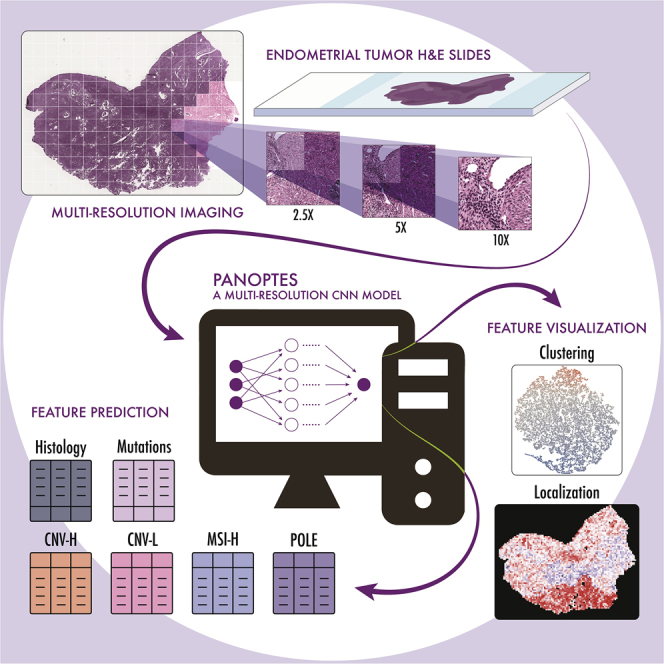

## Teaser

Hong et al. develop and implement a customized multi-resolution deep convolutional neural network that predict molecular subtypes and 18 common gene mutations in endometrial cancer based on digitized H&E-stained pathological images. The models learn human interpretable and generalizable features, indicating potential clinical application without sequencing analysis.

## Introduction

Endometrial cancer is the most common type of gynecologic cancer among women around the world, with an increasing occurrence and mortality.^3^, ^4^, ^5^, ^6^ In the United States, it is one of the top 5 leading cancer types, with 52,600 new cases reported in 2014, which increased to 61,880 in 2019.^5^, ^6^, ^7^ Globally, endometrial cancer caused ∼42,000 women’s deaths in 2005, and this annual mortality count estimate drastically increased to 76,000 in 2016.^3^^,^^4^ The 5-year survival rate, depending on the study cohort, ranges from 74% to 91% for patients without metastasis.^5^

Clinically, endometrial carcinomas are stratified based on their grade, stage, hormone receptor expression, and histological characteristics.^8^ Histological classification reflects tumor cell type and informs the choice of surgical procedure and adjuvant therapy. The majority of endometrial cancer cases exhibit either endometrioid (70%–80% of cases) or serous (10% of cases) characteristics.^9^ Patients with serous subtype tumors have a lower 5-year survival rate due to more frequent metastases and a higher risk of recurrence^4^ Thus, it is critical to determine the subtypes to determine patients’ individualized treatment plans and to assess prognosis.^3^^,^^10^ Histological subtype is determined by pathologists after thorough examination of hematoxylin and eosin (H&E)-stained tissue sample slides of tumor samples. Endometrioid tumors typically exhibit a glandular growth pattern, while the serous subtype is characterized by the frequent presence of a complex papillary pattern.^11^, ^12^, ^13^ These features are not exclusive for either of the subtypes, however, making histological classification challenging, especially among high-grade cases, even for experienced pathologists and necessitating ancillary subtyping criteria.^1^^,^^4^^,^^14^^,^^15^

The Cancer Genome Atlas (TCGA) introduced a set of criteria that classify endometrial carcinoma into four molecular subtypes, namely polymerase ε (POLE) ultra-mutated, high microsatellite instability (MSI-high) hypermutated, copy-number low (CNV-L), and copy-number high (CNV-H), based on their mutation characteristics, copy-number alterations, and microsatellite instability. This molecular classification standard has been gaining popularity among pathologists and clinicians in recent years. Among these four subtypes, patients with the CNV-H subtype, which includes serous carcinomas and a subset of high-grade endometrioid cancers, had the worst outcomes based on progression-free survival.^1^ Exome sequencing also revealed a panel of genes differentially mutated across the four molecular subtypes, many of which have been shown to play significant roles in endometrial carcinoma tumorigenesis and proliferation and can be targets of individualized therapies.^16^^,^^17^ For example, most patients in the CNV-H subtype are *TP53* mutated but *PTEN* wild type. Determining the molecular subtyping and single-gene mutations can provide insights that complement and refine the histological classification, but the availability of this information is limited by the time and cost of sequencing.

Computational approaches for analyzing massive biomedical data have tackled numerous challenges, which accelerate the pace of human health improvement worldwide. Computational pathology, a discipline that involves the application of image processing techniques to pathological data, has been especially benefitted from the advancement of deep learning in recent years.^18^, ^19^, ^20^, ^21^ Convolutional neural network (CNN) models are capable of segmenting cells in histopathology slides and classifying them into different types based on their morphology.^18^^,^^22^ An InceptionV3-based model achieves a high level of accuracy in determining melanoma possibility, exhibiting significant diagnostic potential.^19^ Moreover, successful deep learning models have also been built to predict molecular and genomic features in cancer, such as MSI, immune subtypes, and somatic mutation status, suggesting that machine learning techniques may be able to assist human experts to further exploit clinically relevant information in pathological images.^23^, ^24^, ^25^, ^26^, ^27^, ^28^ In addition, studies have demonstrated that deep neural network models have the potential to capture features across cancer and tissue types.^29^^,^^30^

H&E slides are typically scanned at different resolutions and saved as a single image file. This allows pathologists to examine features of various sizes at the optimal resolution. Here, we designed a customized architecture that we call Panoptes. Panoptes takes advantage of the multi-resolution structure of the H&E image files. We show that models using this architecture can classify common endometrial carcinoma histological subtypes, molecular subtypes, and several critical mutations with decent performance based on H&E images and outcompete existing InceptionResnet models in most top-performing tasks. Using the t-distributed stochastic neighbor embedding (tSNE) dimensionality reduction technique, we extracted and visualized the features learned by models to classify H&E images. These histopathological features were mostly human interpretable, suggesting possibilities of incorporating them into the pathological diagnostic standards. In particular, we confirmed that tumor grade was the major factor that distinguishes the CNV-H molecular subtype from the other three molecular subtypes in the histological endometrioid cases. In addition, the generalizability of models was validated by testing trained models of key predictive tasks on an independent clinical dataset, highlighting their potential clinical use.

## Results

### Data preparation and multi-resolution deep learning-based histopathology image analysis

**Figure 1:**
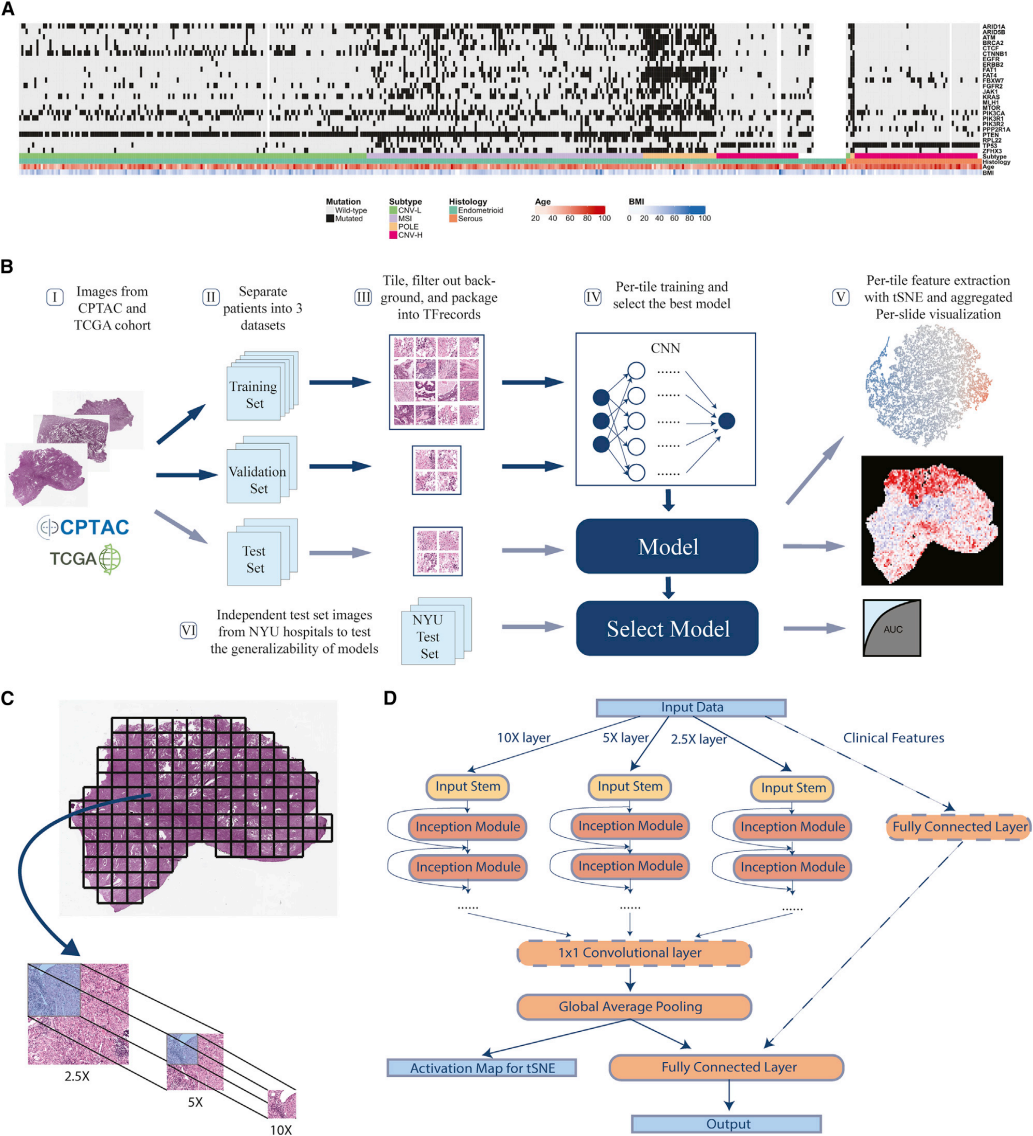
Workflow and Panoptes architecture (A) A total of 456 patients in the cohorts from CPTAC and TCGA with feature annotations. (B) Overall workflow. I, H&E slide images of endometrial cancers were downloaded from databases; II, slides were separated at the per-patient level into a training, validation, and test set; III, slides were cut into 299 × 299-pixel tiles excluding background, and contaminants and qualified tiles were packaged into TFrecord files for each set; IV, training and validation sets were used to train the convolutional neural networks, and the testing set was used to evaluate trained models; V, activation maps of test set tiles were extracted and dimensionally reduced by tSNE to visualize features, while the per-tile predictions were aggregated back into intact slides; VI, an independent test set with samples from NYU hospitals was used to test the generalizability of selected best-performing models. (C) Slides were cut into paired tile sets at 2.5×, 5×, and 10× equivalent resolution of the same region to prepare for Panoptes. (D) Panoptes architecture with optional 1 × 1 convolutional layer and clinical features branch.

We used diagnostic formalin-fixed paraffin-embedded (FFPE) and H&E-stained tumor slides and labels from two public datasets, TCGA and the Clinical Proteomic Tumor Analysis Consortium (CPTAC), to train, validate, and test our models. Later, an independent dataset from samples at New York University (NYU) hospitals were used to test the generalizability and potential clinical capability of select promising trained models. Overall, 496 slides from 456 patients, covered in previous publications^1^^,^^2^ and annotated with subtype and gene mutation information, were included to form a mixed TCGA-CPTAC dataset (Figures 1A and S1A). More than 90% of patients in our cohort had only 1 diagnostic slide (Figure S1B). As many driver gene mutations in endometrial cancer are correlated with histological and molecular subtypes, we validated these correlations to ensure that our cohort was a representative of the patient population (Figure S1C). The general process of training, validation, testing, and visualization followed the workflow in Figure 1B. For each prediction task, cases in the mixed dataset were randomly split into training, validation, and test set, such that slides from the same patient were in only one of these sets. This allowed the test set to be strictly independent to the training process and also made it possible to obtain per-patient level metrics, which could be more useful in the clinical setting. Each task was performed on a different random split of cases stratified with the outcome. Due to the extremely large dimension of the digital H&E slides (Figure S1D), slides were tiled into 299 × 299 pixels and color normalized.

We developed a multi-resolution InceptionResnet-based^31^ CNN architecture, Panoptes, to capture features of various sizes on the H&E slides, which resembles the reviewing strategy of human pathologists. Unlike the conventional CNN architecture, the input of Panoptes is a set of three tiles of the same region on the H&E slide instead of a single tile. The equivalent scanning resolution of tiles in a set is 2.5×, 5×, and 10× so that the higher-resolution tile covers one-fourth of the region in the next lower-resolution tile (Figure 1C). Hence, each grid region at 2.5× resolution can ideally generate 16 tile sets (Figure 1C). Each set of tiles were converted into a single matrix as one sample. Panoptes has three InceptionResnet-based branches, each of which processed the samples with a specific resolution of the same region simultaneously (Figure 1D). These branches worked separately until the third-to-last layer of the architecture, in which inputs from the three branches were concatenated, followed by a global average pooling layer and the final fully connected layer. This design enabled the branches to learn features of different scales. More abstract information from each branch was integrated only at higher levels. We attempted to add an additional 1 × 1 feature pooling convolutional layer before the global average pooling and introduced a fourth branch processing clinical feature, including patients’ age and body mass index (BMI). Compared to conventional CNN, the multi-resolution design of Panoptes can at the same time consider both macro-tissue-level features and minute cellular-level features of the same region, and can therefore capture more comprehensive characteristics of the slides. Moreover, taking the multi-resolution tile sets as input while having a single output and loss function preserves the original spatial information, which makes Panoptes distinct from the simply joining decisions from three separate models trained on tiles of three resolutions. We tried four different Panoptes architectures with and without the clinical feature branch, two types of InceptionResnet, and three types of Inception in this study. InceptionResnet and Inception models were trained on single resolution 10× tiles. Among all of the statistical metrics calculated, we used area under the receiver operating characteristic curve (AUROC) of the test sets as the major metric to evaluate the performance of the models, which is the typical standard in the machine learning field. Precision, recall, sensitivity, specificity, and F1 scores were also considered to evaluate imbalanced prediction tasks. Per-patient level prediction was obtained by taking the mean of the predicted probability (prediction score) of all of the tiles or tile sets belonging to the same patient. The AUROC was then calculated by taking each patient as one sample point. For Panoptes models, one set of tiles was counted as a single tile for the metric calculation purpose since the output from the model was only one prediction score for each set.

### Multi-resolution deep-learning architectures achieved better predictive performance on histopathology images

**Figure 2:**
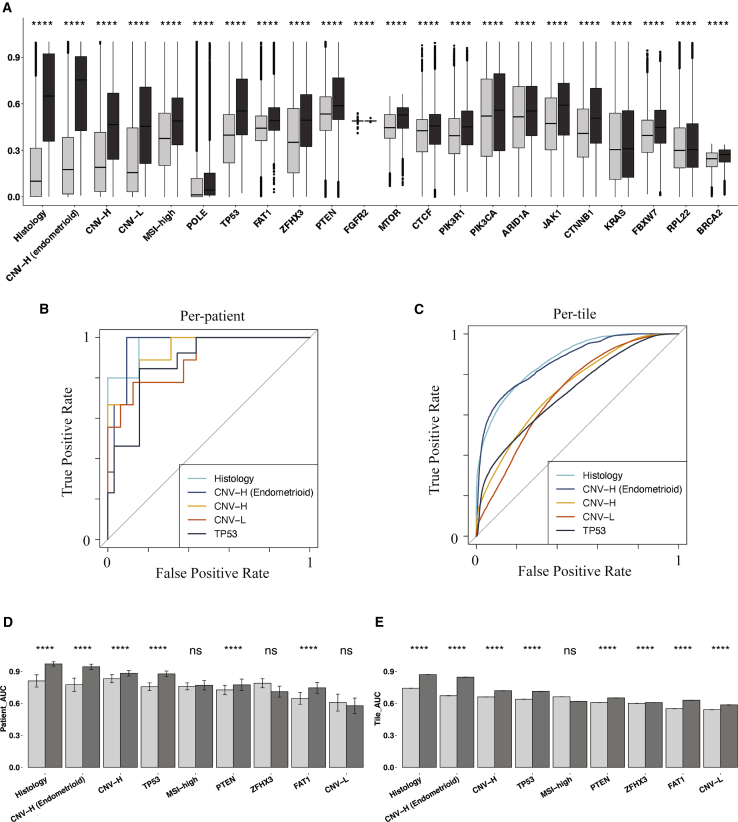
Prediction tasks were statistically successful, with promising results, and Panoptes outcompeted baselines in most of the top-performing prediction tasks (A) Predicted positive probability of tiles with 1-tail Wilcoxon test between true label-positive and -negative groups (black: true label-positive tiles; gray: true label-negative tiles) from models in Table 1. (B and C) ROC curves at per-patient (B) and per-tile (C) level associated with the top 5 prediction tasks in (A). (D and E) Bootstrapped per-patient (D) and per-tile (E) AUROC of InceptionResnetV2 (light) and Panoptes2 (dark) of top 9 tasks in (A) with 1-tail t test.

We trained models to predict histological subtypes, CNV-H subtype from the entire cohort and the endometrioid patients, CNV-L subtype, MSI-high subtype, POLE subtype, and the mutation status of 18 endometrial carcinoma-related genes. We applied five baseline models (InceptionV1, InceptionV2, InceptionV3, InceptionResnetV1, and InceptionResnetV2) and four versions of multi-resolution models (Panoptes1 to -4) on all of the tasks (Figures S2A and S2B). The same data splits were used for all of the models of the same predictive tasks to have fair comparisons among models with different architectures. The best-performing architectures for each of the prediction tasks and their corresponding AUROC with 95% confidence intervals (CIs) are shown in Table 1. Tasks with per-patient AUROC < 0.6 were not listed. We performed one-tail Wilcoxon tests on prediction scores between positively and negatively labeled tiles for the results in Table 1, which all showed significant differences (Figure 2A). Therefore, the prediction scores of true-label-positive tiles were significantly higher than those of true-label-negative tiles, demonstrating that these models were able to distinguish tiles in the test sets.

**Table 1:** AUROC of the best models for each task

	Best architecture	Per-patient AUROC	Per-tile AUROC
Histology	Panoptes2	**0.969 (0.905––1)**	**0.870 (0.866–0.874)**
CNV-H from endometrioid	Panoptes1	**0.958 (0.886––1)**	**0.864 (0.859–0.870)**
CNV-H	Panoptes4	**0.934 (0.851–1)**	0.731 (0.728–0.734)
POLE	Multi-model system	**0.890 (0.821–0.960)**	0.691 (0.683–0.700)
CNV-L	Panoptes1	**0.889 (0.755–1)**	0.710 (0.705–0.716)
TP53	Panoptes2	**0.873 (0.768–0.977)**	0.713 (0.709–0.717)
FAT1	Panoptes2 with clinical	**0.835 (0.666–1)**	0.639 (0.635–0.642)
MSI-high	InceptionResnetV1	**0.827 (0.705–0.948)**	0.638 (0.635–0.641)
ZFHX3	InceptionResnetV1	**0.824 (0.689–0.959)**	0.637 (0.634–0.640)
PTEN	InceptionV2	**0.781 (0.579–0.984)**	0.623 (0.620–0.627)
FGFR2	Panoptes4 with clinical	**0.755 (0.540–0.970)**	0.550 (0.545–0.554)
MTOR	Panoptes1	0.724 (0.496–0.951)	0.674 (0.670–0.678)
CTCF	Panoptes4	0.724 (0.518–0.931)	0.571 (0.568–0.575)
PIK3R1	InceptionResnetV1	0.702 (0.524–0.880)	0.596 (0.593–0.599)
PIK3CA	Panoptes4	0.689 (0.532–0.847)	0.526 (0.523–0.530)
ARID1A	InceptionResnetV2	0.683 (0.513–0.853)	0.542 (0.538–0.545)
JAK1	Panoptes2 with clinical	0.662 (0.410–0.940)	0.612 (0.605–0.618)
CTNNB1	InceptionResnetV2	0.648 (0.439–0.858)	0.619 (0.616–0.622)
KRAS	Panoptes2 with clinical	0.638 (0.404–0.871)	0.515 (0.510–0.519)
FBXW7	InceptionV3	0.629 (0.366–0.892)	0.606 (0.602–0.609)
RPL22	InceptionV3	0.632 (0.395–0.868)	0.517 (0.512–0.522)
BRCA2	InceptionResnetV1	0.613 (0.318–0.908)	0.624 (0.620–0.629)

Bootstrapped 95% confidence intervals (CIs) are listed in parentheses. AUROCs > 0.75 are listed in bold.

Based on the AUROC scores, we observed that Panoptes models were the best architectures in six of the top seven prediction tasks (Figures 2B and 2C; Table 1). We also observed that Panoptes performed better than Inception and InceptionResnet models for most of the tasks (Figures S2A and S2B). To validate that Panoptes performed better than InceptionResnet, we conducted a one-tail t test on AUROC performance of the top nine prediction tasks between the Panoptes models and their corresponding InceptionResnet models. Panoptes2, which was the best Panoptes architecture in most of the tasks, showed a significantly higher AUROC than the corresponding InceptionResnet2 in six prediction tasks at the per-patient level and eight at the per-tile level (Figures 2D and 2E). Similarly, Panoptes1 had a significantly higher AUROC than InceptionResnet1 in five prediction tasks at the per-patient level and eight at the per-tile level (Figures S3A and S3B).

To evaluate the effectiveness of adding an additional 1 × 1 convolutional layer between the concatenation of branches and the global average pooling, we performed a one-tail t test between Panoptes1 and Panoptes3 as well as Panoptes2 and Panoptes4. However, only four tasks at the per-patient level and six tasks at the per-tile level showed significant p values between Panoptes2 and Panoptes4 (Figures S3C and S3D). Similar results were observed between Panoptes1 and Panoptes3, in which only one per-patient level task and four per-tile level tasks showed a significant difference. By applying the same test to Panoptes with and without clinical feature branch models, most of the tasks were not statistically significant, with an example of Panoptes2 having four significant tasks at the per-patient level and five at the per-tile level (Figures S3E and S3F). In summary, our multi-resolution architecture Panoptes outperformed InceptionResnet in analyzing endometrial cancer H&E slides in various prediction tasks. The effectiveness of the additional convolutional layer and the integration of patients’ age and BMI through a fourth branch was not found to be significant, however.

### Accurate predictions of histological and molecular subtypes

Panoptes2 achieved a 0.969 (95% CI: 0.905–1) per-patient level AUROC in classifying samples into endometrioid or serous histological subtypes, with an F1 score of 0.75. The precision was 1 and the recall was 0.6, respectively, at the per-patient level. Panoptes models were in the leading positions, followed by InceptionV3 and InceptionV2, all of which had per-patient AUROCs > 0.9. For molecular subtyping tasks, we applied all architectures on four binary tasks, each aimed at predicting one molecular subtype versus all others. Panoptes1 achieved a per-patient AUROC of 0.934 (95% CI: 0.851–1) in predicting CNV-H, while all other Panoptes models achieved an AUROC > 0.88, outcompeting the baseline models by 5.8%–23.3%. The best F1 score was 0.8, with precision of 0.727 and recall of 0.889, respectively. This model also achieved a sensitivity of 0.889 and a specificity of 0.906 when using 0.5 as the cutoff point of prediction scores. For the CNV-L subtype classification, Panoptes1 achieved a per-patient AUROC of 0.889 (95% CI: 0.755–1), outcompeting the baseline models by 12%. The F1 score was 0.75, with precision of 0.857 and recall of 0.667. For MSI-high, the best per-patient AUROC was 0.827 (95% CI: 0.705–0.948) and F1 score was 0.615. A POLE subtype classification model achieved a per-patient AUROC of 0.681 (95% CI: 0.499–0.863).

Although most CNV-H cases are of the serous subtype, a portion of high-grade endometrioid cancers are also classified as CNV-H. To further assess whether machine learning models could capture the heterogeneity within this histological subtype, we trained models to predict CNV-H status in endometrioid samples. The Panoptes1 architecture was able to achieve a per-patient AUROC of 0.958 (95% CI: 0.886–1) and an F1 score of 0.667 on this task, suggesting that the model used features that were not strongly associated with the histological subtype to predict the molecular subtype. All Panoptes models also outcompeted baseline models in this task. In addition, we trained models to predict the mutation status of 18 driver genes. Panoptes2 was able to predict a *TP53* mutation with a per-patient AUROC of 0.873 (95% CI: 0.768–0.977) and an F1 score of 0.56. *FAT1* mutation was predicted using Panoptes2 (with the clinical feature branch) with a per-patient AUROC of 0.835 (95% CI: 0.666–1) and an F1 score of 0.545. Other gene mutations, including *ZFHX3*, *PTEN*, *FGFR2*, *MTOR*, *CTCF*, and *PIK3R1*, were also predicted with a per-patient AUROC > 0.7. The full statistical metrics for all of the prediction models are in Table S1.

### Feature extraction and whole-slide visualization revealed correlations and differences between histological and molecular features

**Figure 3:**
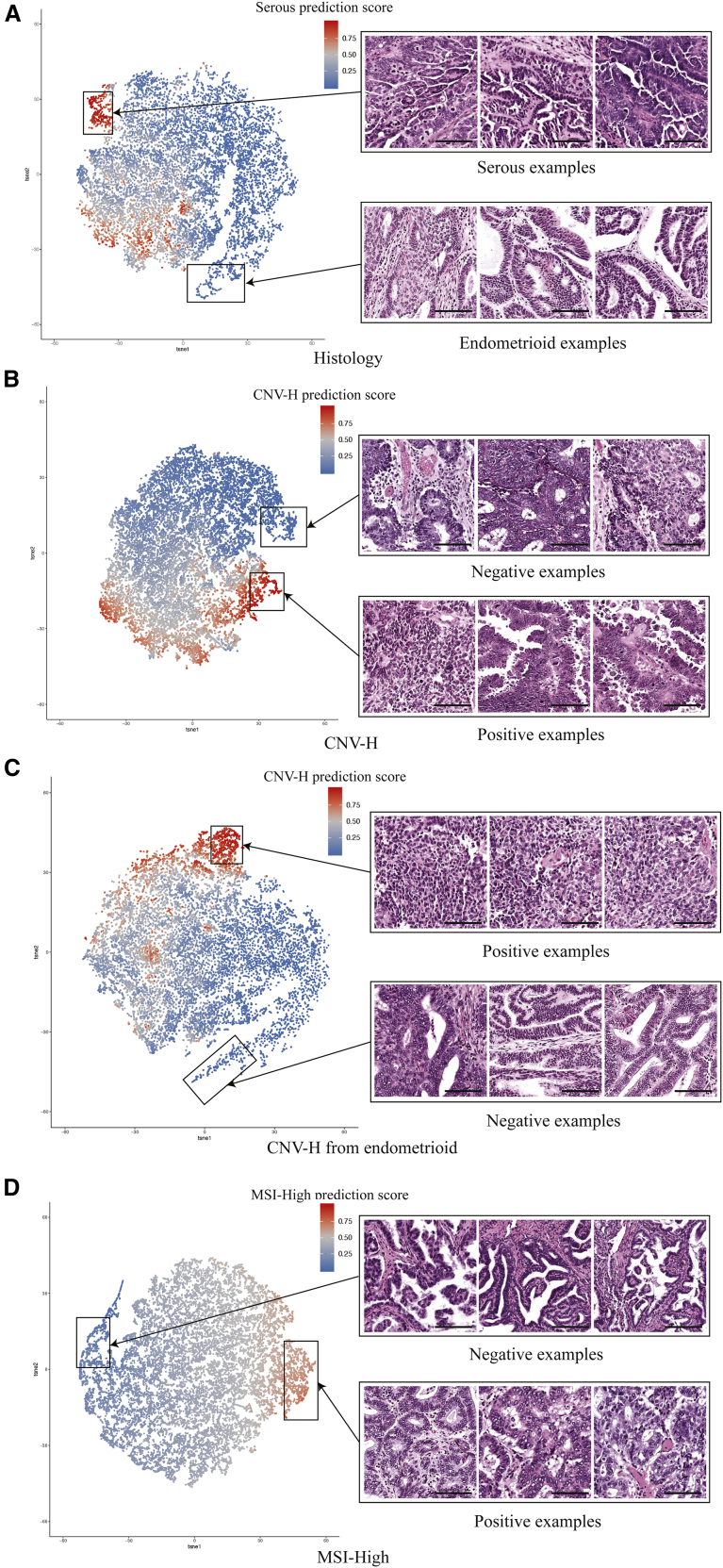
Extraction and visualization of features learned by the models with tSNE Each point represents a tile and is colored according to its corresponding positive prediction score. Scale bars represent 100 μm. (A) Histologically serous and endometrioid features from a Panoptes1 model. (B) CNV-H-positive and -negative features from a Panoptes4 model. (C) CNV-H-positive and -negative features in the histologically endometrioid samples from a Panoptes1 model. (D) MSI-high positive and negative features in the histologically endometrioid samples from a Panoptes3 with clinical features model.

To visualize and evaluate features learned by the models for each task, we extracted the activation maps before the final fully connected layer of the test set tiles. The activation maps of 20,000 tiles were then randomly sampled for each task. These activation maps were dimensionally reduced and displayed on two-dimensional (2D) tSNE plots, in which each dot represents a sampled tile and was colored according to the positive prediction scores (Figure 3). As we expected, tiles were generally clustered by their predicted groups. By replacing dots with the original input tiles of different resolutions, we were able to discover features that correlated with the predictions corresponding to the specific histological or molecular classification task. For example, features of predicted histologically serous and endometrioid were drastically different (Figure 3A). In the cluster with high prediction scores of serous subtypes, we observed typical serous carcinoma features, such as high nuclear grade, papillary growth pattern, elevated mitotic activity, and slit-like spaces. Tiles in the cluster of predicted endometrioid cases showed low nuclear grade, glandular growth pattern, cribriform architecture, and squamous differentiation. Myometrium and other non-tumor tissue tiles were located in the middle of the tSNE plot, with prediction scores between 0.4 and 0.6. These observations suggested that our models were able to focus on the tumor regions of H&E slides and make histological subtype predictions based on features that were also recognized by human experts in pathology.

The features learned by molecular subtype prediction models were also revealed with the same feature extraction method. We noticed that in the CNV-H prediction model, two distinct subgroups were recognized in the predicted CNV-H cluster, associated with histological serous and high-grade endometrioid subtypes, respectively (Figure 3B). The predicted CNV-H serous tiles mostly showed high nuclear grade, gland formation, and elevated mitotic activity, while the predicted CNV-H high-grade endometrioid tiles exhibited solid growth pattern and focal glandular differentiation. In contrast, in the non-CNV-H cluster, tiles were mostly low-grade endometrioid carcinoma with low nuclear grade, gland formation, and squamous differentiation (Figure 3B). To confirm that the tumor grade was the major factor to distinguish CNV-H molecular subtype in endometrioid samples, we unveiled the features learned by the CNV-H prediction model trained only on endometrioid images (Figure 3C). As we expected, high-grade endometrioid carcinoma tiles were observed mostly in the CNV-H cluster, leaving the low-grade tiles in the non-CNV-H cluster. In both of these CNV-H models, the ambiguous regions were mostly occupied by non-tumor tissue. We also visualized the major pattern learned by the model to distinguish MSI-high subtype images from others (Figure 3D). Tiles in the MSI-high cluster were mostly low-grade endometrioid carcinomas with gland formation, tumor-infiltrating lymphocytes, and peritumoral lymphocytes, consistent with the observation that the heavy mutation load of MSI-high tumors led to high immunogenicity and a host immune response.^32^^,^^33^

In addition to the subtypes, patterns related to some mutations were revealed. A *PTEN*-mutated cluster mostly contained tiles of low-grade endometrioid carcinomas with gland formation and low nuclear grade (Figure S4A), while *TP53*-mutated tiles were generally serous carcinomas with high nuclear grade and abundant tufting and budding (Figure S4B). Furthermore, low-grade endometrioid carcinoma tiles with gland formation, low nuclear grade, and abundant tumor-infiltrating lymphocytes were present in the *ZFHX3*-mutated cluster, while those with much fewer lymphocytes were in the wild-type cluster (Figure S4C). High-grade endometrioid carcinoma tiles with diffuse solid growth and low nuclear grade were depicted in the *FAT1*-mutated cluster, while low-grade endometrioid carcinomas with gland formation, low nuclear grade, and cribriform architecture were in the wild-type cluster (Figure S4D). These findings may result from the correlation between mutation status and histological or molecular subtypes described above, as *PTEN* and *TP53* mutations were mainly found in endometrioid and serous subtypes, respectively, while *ZFHX3* and *FAT1* mutation status showed correlation with the heavily mutated MSI-high and *POLE* molecular subtypes (Figure S1C).

**Figure 4:**
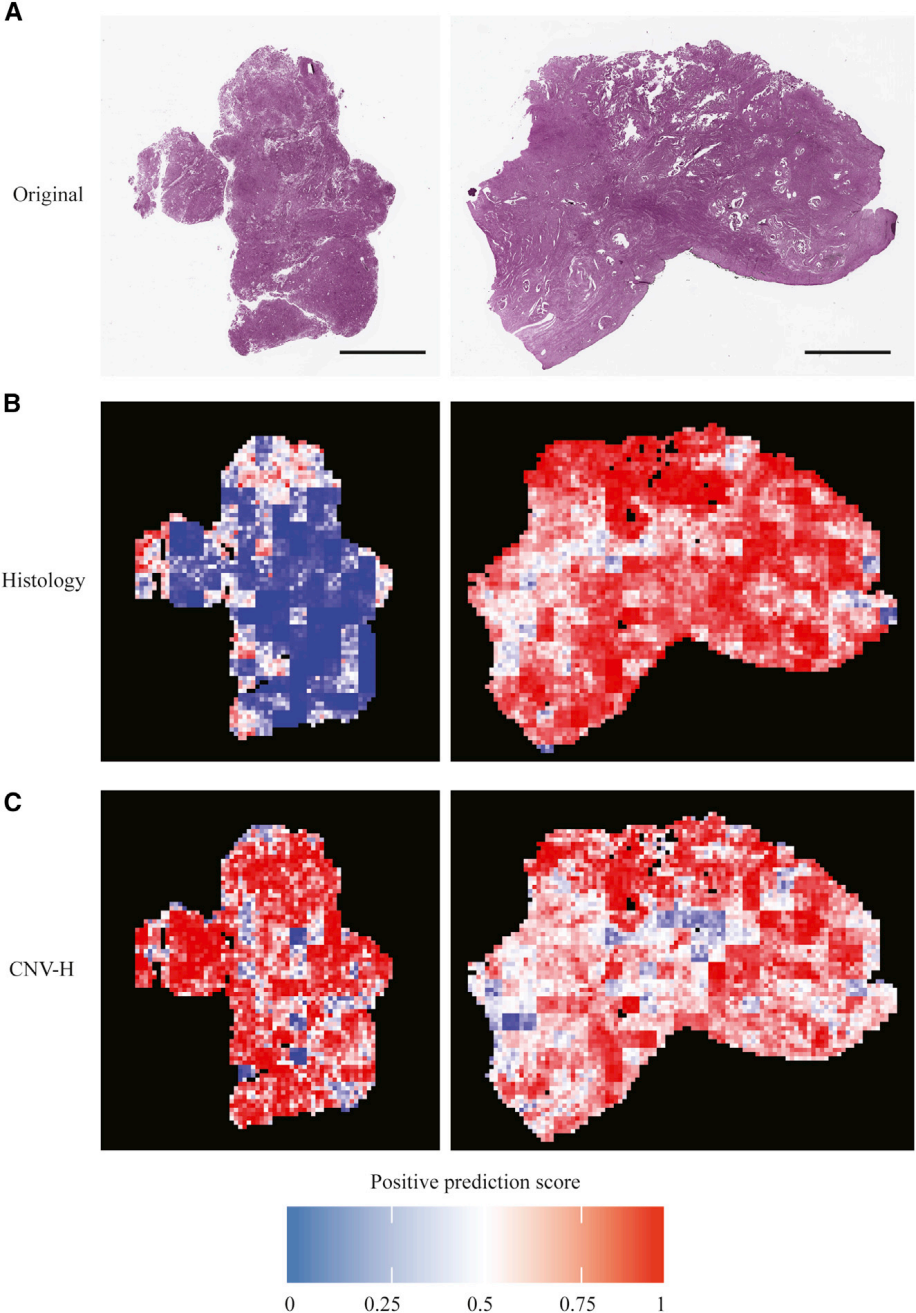
Whole-slide predictions showing that some features of determining histological subtype and CNV-H are distinct (A) The first slide is from a CNV-H but histologically endometrioid case, while the second slide is from a CNV-H and serous tumor. Scale bars represent 5,000 μm. (B) Whole-slide histology prediction of examples in (A) from a Panoptes2 model, with hotter regions being predicted were more serous, while cooler regions were more endometrioid. (C) Whole-slide CNV-H prediction of examples in (A) from Panoptes1 (first example) and Panoptes4 (second example) models, with hotter regions being predicted were more CNV-H.

In addition, we were interested in visualizing the spatial distribution of features on the whole-slide level. Prediction of tiles from the test sets were aggregated back to the size of the original slides in the form of heatmaps, in which hotter tiles corresponded to higher positive prediction scores. Whole-slide visualization revealed that our models tended to have extreme prediction scores on tumor regions instead of non-tumor tissues such as myometrium (Figure S5). The first slide in Figure 4 was from an endometrioid and CNV-H case, while the second slide was from a serous and CNV-H case. Models correctly predicted both tasks for the two slides. By comparing the prediction of histological subtypes and CNV-H, we found that the models were focused on different yet related features in these two prediction tasks. In the first slide, the areas predicted to be endometrioid were largely classified as CNV-H, while in the second slide, most of the areas predicted as serous were also classified as CNV-H. This suggested that although most CNV-H samples were histologically serous, our models relied on some additional features other than those of histological subtypes—likely tumor grade—to separate CNV-H samples from endometrioid samples.

### Generalizability and potential clinical capability of the models

**Figure 5:**
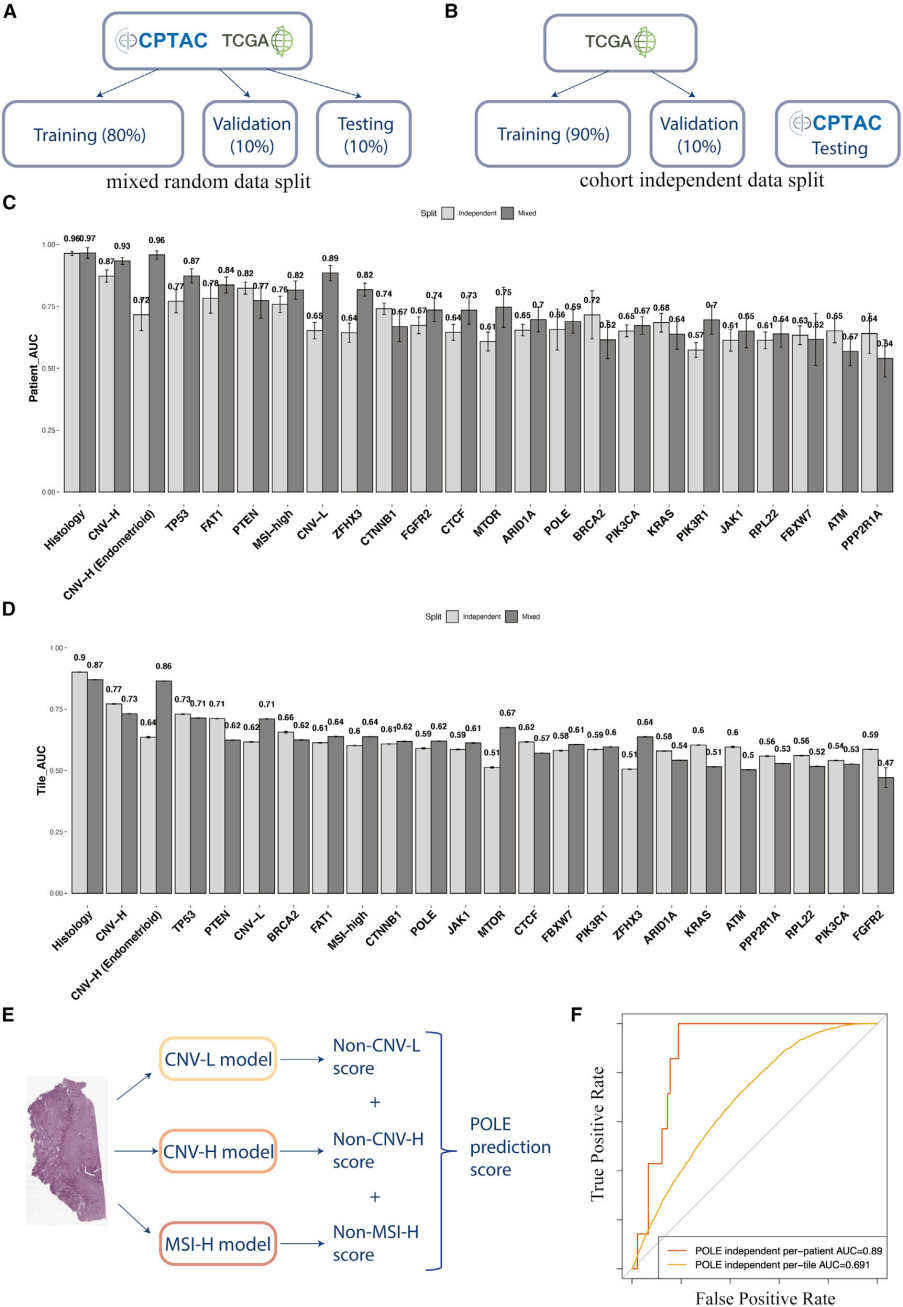
Comparisons of AUROC between the best models in mixed random split trials and cohort independent split trials and multi-model system for better POLE subtype classification (A) Mixed random data split demonstration. (B) Cohort independent data split demonstration. (C and D) Per-patient (C) and per-tile (D) level AUROC of the best-performing models in each task with mixed random data split (dark) and the cohort independent data split (light). Error bars indicate bootstrapped confidence interval. (E) Multi-model system to indirectly predict POLE molecular subtype. (F) ROC curves at per-patient and per-tile level of multi-model POLE classification system.

To ensure the generalizability of models, especially those with Panoptes architectures, we adopted cohort independent data split in addition to mixed random data split and retrained all of the predictive models from scratch (Figures 5A and 5B). The AUROC of the CPTAC independent test set indicated that Panoptes-based models still showed better performance than the baseline models in general (Figures S2C and S2D). The best-performing models based on the CPTAC independent test set were compared side by side with the best models in mixed random split trials (Figures 5C and 5D). A Panoptes4 model achieved an AUROC of 0.962 (95% CI: 0.926–0.999), with an F1 score of 0.696 at the per-patient level in predicting histological subtypes, which were similar to the best model on the mixed random data split. In the CNV-H molecular subtype prediction task, a Panoptes3 model showed an AUROC of 0.87 (95% CI: 0.753–0.987), with an F1 score of 0.667 at the per-patient level. Slightly lower performances were also observed in prediction tasks using cohort independent data split at the per-patient level, including MSI-high, POLE, *TP53*, and *FAT1*. However, higher statistical metrics were observed in some prediction tasks, such as *PTEN*, *KRAS*, *BRCA2*, and *CTNNB1*. Interestingly, even though the per-patient level metrics were lower in the cohort independent data split trials than in the mixed data split trials for some prediction tasks (CNV-H, *TP53*, *CTCF*), their per-tile level metrics were higher. In addition, we compared Panoptes-based models’ performance side by side in mixed random split trials and cohort independent split trials and the results were similar to the best-performing models’ comparisons (Figure S6). The full table of statistical metrics of the test set in the cohort independent split trials are in Table S2.

The best-performing model for the POLE subtype classification with cohort independent data split achieved a per-patient level AUROC of 0.679 (95% CI: 0.42–0.939), lower than the other three molecular subtype classification models. To improve the POLE classification, a multi-model system was built by aggregating negative prediction scores of the other three molecular subtypes (Figure 5E). A system consisting of Panoptes2 models of CNV-H and CNV-L and the InceptionResnetV1 model of MSI-high with cohort independent data split achieved a per-patient AUROC of 0.89 (95% CI: 0.821–0.96) and a per-tile AUROC of 0.691 (95% CI: 0.683–0.7) for the POLE subtype (Figure 5F). The full table of statistical metrics of the POLE multi-model classification systems are in Table S3.

**Figure 6:**
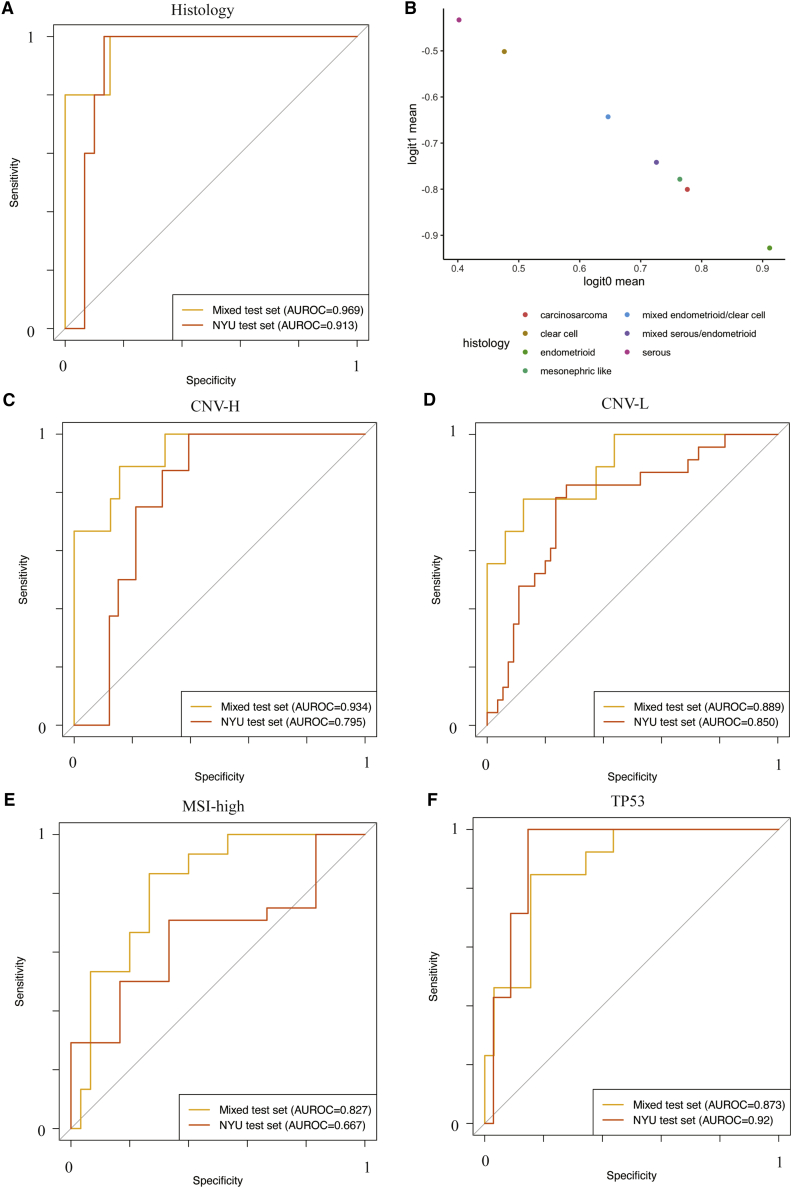
ROC curves and AUROC of the best-trained models of key tasks showed promising predictive power on the independent clinical dataset (A) Trained Panoptes2 histological subtype predictive model on the mixed TCGA and CPTAC held-out test set and the NYU test set. (B) Mean prediction logits by histology from histological subtype predictive model of NYU test set samples. (C) Trained Panoptes4 CNV-H subtype predictive model on the mixed TCGA and CPTAC held-out test set and the NYU test set. (D) Trained Panoptes1 CNV-L subtype predictive model on the mixed TCGA and CPTAC held-out test set and the NYU test set. (E) Trained InceptionResnetV1 MSI-high subtype predictive model on the mixed TCGA and CPTAC held-out test set and the NYU test set. (F) Trained Panoptes2 *TP53* mutation predictive model on the mixed TCGA and CPTAC held-out test set and the NYU test set.

To further illustrate potential clinical capability, we retrieved another retrospective independent test set consisting of 137 deidentified FFPE H&E slides from 41 patients at NYU hospitals to test the trained models of some prediction tasks, including histological subtypes, CNV-H, CNV-L, MSI-high, and *TP53* mutation (Table S4). This clinical dataset was more diversified histologically as it contained not only serous and endometrioid samples, but also samples of rare histological subtypes, including clear cell, carcinosarcoma, mesonephric-like, and mixed histology. As genomic sequencing data were not available for this cohort, we used immunohistochemically identified *P53* overexpression as a surrogate label for TP53 aberration. The Panoptes2 histological subtype predictive model trained on the mixed TCGA-CPTAC dataset achieved a per-patient level AUROC of 0.913 (95% CI: 0.816–1) on the NYU test set, with an F1 score of 0.714 (Figure 6A). Notably, this model was the best-performing model based on both the mixed held-out test set and the NYU test set. Although the rare histological subtype samples were excluded in statistically metrics calculation, their mean prediction logits generally lay between serous and endometrioid samples, suggesting that they could be linearly separable by setting up appropriate thresholds (Figure 6B). The Panoptes4 CNV-H predictive model, which was the best-performing one according to the mixed test set, achieved an AUROC of 0.795 (95% CI: 0.66–0.931) on the NYU test set, which was lower than 8 other trained models with AUROCs ranging from 0.818 to 0.894 (Figure 6C; Table S4). Due to the lack of samples, some statistical metrics, such as the per-patient level AUROC, could not be calculated for the CNV-H in endometrioid predictive models. The per-tile level AUROC of the Panoptes2 model on the NYU test set was 0.919 (95% CI: 0.911–0.926), similar to its performance on the mixed test set (Tables S1 and S4). The Panoptes1 CNV-L predictive model achieved a per-patient AUROC of 0.85 (95% CI: 0.732–0.968) on the NYU test set, similar to its performance on the mixed test set, which was the best among all of the models (Figure 6D; Table S1). The best-performing MSI-high predictive model on the mixed test set was also the best one on the NYU test set (Figure 6E). Interestingly, the Panoptes2 *TP53* aberration prediction model achieved a higher per-patient level AUROC on the NYU test set (0.92; 95% CI: 0.836–1) than on the mixed test set (Figure 6F). This was likely due to the fact that the *TP53* aberration determined at the expression level was easier for the model to detect, further supporting our conclusion that the model recognized morphological manifestations of genetic aberration. In general, the performance of TCGA-CPTAC mixed data split trained models on the NYU test set were comparable to their performance on the mixed held-out test set in these tasks, suggesting that these models are generalizable on independent clinical samples. We also tested models training only on TCGA samples (cohort independent data split) on the NYU test set, and the performances were mostly slightly lower than the mixed data split trained performances (Table S4).

## Discussion

Our study introduced a multi-resolution InceptionResnet-based CNN architecture, Panoptes, which was able to predict endometrial cancer histological and molecular subtypes and the mutation status of critical genes based on H&E slides and generalized well on independent test sets. The AUROC of classifying endometrioid and serous histological subtypes by our best architecture model was 0.969 (95% CI: 0.905–1). Moreover, the models can distinguish the most lethal molecular subtype, CNV-H, with exceptionally high accuracy (AUROC 0.934). It is worth noting that our models can also precisely identify the CNV-H samples from a histologically endometrioid carcinoma (AUROC 0.958), which is one of the more controversial and complex patient subgroups in endometrial cancer subtyping. In addition to the CNV-H, we were able to predict other molecular features with acceptable performance, which are not possible for pathologists to determine without ancillary studies, such as sequencing or immunohistochemistry. These include the CNV-L molecular subtype (AUROC 0.889), the DNA-mismatch repair deficiency-related MSI-high molecular subtype (AUROC 0.827), the mutation of the CNV-H signature gene *TP53* (AUROC 0.873), as well as *PTEN* (AUROC 0.781), *FAT1* (AUROC 0.835), and *ZFHX3* (AUROC 0.824). Although the direct predictive model for the POLE molecular subtype did not achieve promising results due to insufficient training samples, an indirect multi-model system approach allowed us to classify the POLE subtype effectively (AUROC 0.89). Statistical analyses proved the success of our prediction tasks. In addition, we tested and showed that our multi-resolution Panoptes-based models performed significantly better than InceptionResnet-based models in most of our prediction tasks. We implemented two modifications to Panoptes, but we did not observe significant improvements in performance for the majority of the tasks. We discovered critical features to distinguish subtypes and mutations, particularly the tumor grade in determining CNV-H cases from non-CNV-H samples. We tested the generalizability of our models by applying them to an independent dataset of samples from NYU hospitals for some of the most promising prediction tasks and showed that the model generalizes well to clinical independent data. In addition, even though the NYU test set contained some samples of rare histological subtypes, their prediction logits suggested that they could be separated by our models with additional simple thresholds. Moreover, we observed a noticeable decrease in the performance of the MSI-high prediction model on the NYU test set, which is similar to previous studies when applying models to independent clinical images.^29^^,^^30^ We believe that more MSI-high training samples would be essential to allow the model to capture the full heterogeneity of MSI tumors and be more generalizable. We were not able to conduct generalizability tests for all of the prediction tasks using this NYU deidentified clinical dataset due to the lack of relevant sequencing information. Instead, for other prediction tasks, we retrained the models with only TCGA samples and tested on the CPTAC samples to prove their generalizability. Although slightly lower performance levels were observed in the CPTAC-only testing trials for some prediction tasks, we believe that it was most likely caused by a smaller and less diversified TCGA-only training set. The differences in feature distributions among samples in the training set and the independent test sets could also be factors that affected the models’ performance. Previous publications on pan-cancer level imaging models trained on TCGA dataset with the transfer learning technique reported AUROCs of 0.80 (95% CI 0.78–0.87) in the TP53 mutation and 0.82 (95% CI 0.76–0.89) in the PTEN mutation prediction.^29^^,^^30^ On the two common tasks, our end-to-end multi-resolution model achieved per-patient level AUROCs of 0.873 (95% CI 0.768–0.977) in TP53 mutation and 0.781 (95% CI 0.579–0.984) in PTEN mutation prediction. However, it remained unclear which model performed better on these specific tasks, given the difference of the scope of the studies and the datasets.

Examining H&E slides is still the most widely used technique for pathologists to confirm endometrial cancer histological subtypes in the clinical setting. Our models showed great potential in assisting pathologists making decisions and improving diagnostic accuracy. Given that most H&E slides can be tiled into <5,000 tile sets (Figures S1E–S1G), with a processing speed of 22 tile sets per second on a Quadro P6000 GPU, our models can analyze a slide within 4 min. This means that these models can work simultaneously with pathologists to serve as references. We have shown that the model used human-interpretable features to perform histological and molecular classification tasks. With whole-slide visualization, the reassembled per-tile predictions can provide a thorough examination of the H&E slide and a detailed layer containing potential hotspot features, which may also include regions that could be neglected by pathologists. However, due to the time-consuming H&E slide scanning and tiling processes, multiple optimizations need to be implemented before the system can be deployed in practice.

Overall, we demonstrated that our multi-resolution CNN architecture, Panoptes, can be developed into a practical tool to assist pathologists’ classifying endometrial cancer histological subtypes and, more important, to provide additional information about patients’ molecular subtypes and mutation status in a much more rapid fashion and without the need for sequencing. In addition to per-patient level prediction, the model would also be able to highlight regions with human-interpretable features on the slide. Moreover, it remains possible that our models have learned visual patterns correlating with molecular features that were not previously annotated by human experts and, thus, requires further investigation. We believed that these artificial intelligence (AI)-based models have the potential to reveal associations that human experts traditionally would not focus on, such as morphological features related to driver mutations. From another perspective, these patterns from the H&E slides may be incorporated into the current standards of histological pathology and contribute to improved prognosis and treatment of endometrial carcinoma in the future. Predictive models are not able to distinguish causal effects from statistical correlations, and one must be extremely careful with interpretations of the model outputs. Even sequencing efforts would suffer from the same problem, that the presence of a certain mutation does not necessarily mean that the mutated gene is driving the tumorigenesis. However, we may be able to take advantage of the predictive power of our neural network models by using carefully chosen labels. For example, as a well-known driver gene in endometrial cancer, accurate and rapid prediction of TP53 mutation status based on cost-efficient histopathological images would provide very helpful information in a clinical setting.

Our future plan includes refining the Panoptes architecture to improve the overall performance. Quantification of features could also be added. The models need to be trained on more diversified data to meet the more stringent criteria for real world clinical application. We plan to develop a more advanced graphical user interface (GUI) in a fast and user-friendly way, which we are hoping to deploy and test in a pathologist’s clinical practice. We also plan to train Panoptes-based models to predict features in other types of cancers, such as glioblastoma, melanoma, and lung adenocarcinoma, and it would be very interesting to see how Panoptes performs and what features it captures in these tasks.

### Limitations of the study

Both histological and molecular features’ labels of TCGA and CPTAC samples have been validated by many scientists and clinicians before and after the publication of their studies. The NYU test set labels were also validated by pathologists. However, as tile labels were assigned at the per-patient level, within-slide heterogeneity would still lead to noise in the true labels, such that features in a local region may not match the characteristics of the assigned classification. The performance can be further improved if more detailed annotations exist on the slides. From the visualization results, we noticed that our models were more likely to give non-tumor tissue tiles ambiguous prediction scores (0.4–0.6). Therefore, building a segmentation model or to set up a threshold to exclude these irrelevant non-tumor tissue, such as myometrium, may also significantly enhance the overall performance of our models. Although the datasets we used cover a variety of endometrial carcinoma samples, it may not reflect the full pathological diversity and feature distribution of endometrial cancer. More diversified training sets could improve the robustness of the models and, ideally, boost the prediction performance.

## STAR★Methods

### Key resources table

**Table:** 

REAGENT or RESOURCE	SOURCE	IDENTIFIER
**Deposited data**		

Panoptes	This paper	https://github.com/rhong3/Panoptes
Panoptes implementation	This paper	https://github.com/rhong3/CPTAC-UCEC
Genomic Data Commons Data Portal	National Cancer Institute	https://portal.gdc.cancer.gov
TCIA	The Cancer Imaging Archive	https://fairsharing.org/FAIRsharing.jrfd8y
cBioPortal	Cerami et al., 2012^34^; Gao et al., 2013^35^	https://www.cbioportal.org

**Software and algorithms**		

Panoptes Python3 package	This paper	https://pypi.org/project/panoptes-he/
Tensorflow	N/A	https://www.tensorflow.org
Inception	Szegedy et al., 2015^36^	https://github.com/google/inception
InceptionResNet	Szegedy et al., 2017^31^	https://github.com/tensorflow/models/tree/master/research/slim/nets
Keras	N/A	https://keras.io

**Other**		

NVIDIA Tesla V100 GPU	NYU Langone Health BigPurple HPC Cluster	https://med.nyu.edu/research/scientific-cores-shared-resources/high-performance-computing-core
NVIDIA Tesla P40 GPU	NYU HPC Cluster	http://wikis.nyu.edu/Shibboleth.sso/Login?target=https%3A%2F%2Fwikis.nyu.edu%2Fpages%2Fviewpage.action%3FspaceKey%3DNYUHPC%26title%3DHigh%2BPerformance%2BComputing%2Bat%2BNYU

### Resource availability

#### Lead contact

Further information and requests for resources should be directed to and will be fulfilled by the Lead Contact, David Fenyö (David@FenyoLab.org).

#### Materials availability

This study did not generate new unique reagents.

### Experimental model and subject details

The study was entirely computational and did not involve human subjects as it obtained neither data through intervention or interaction with living individuals nor identifiable private information. An NYU Grossman School of Medicine IRB self-certification form was documented and the study complied with institutional requirements at all time. 392 diagnostic slides from 361 endometrial cancer patients in TCGA cohort were downloaded from the NCI-GDC Data Portal. These samples were published in the TCGA pan-cancer atlas. Demographic, genomic, and other clinical features associated with these samples were downloaded from the cBioPortal and the original TCGA endometrial cancer publication supplements.^1^ 107 diagnostic slides from 98 endometrial cancer patients in CPTAC cohort were downloaded from The Cancer Imaging Archive (TCIA). Demographic, genomic, and other clinical features of these patients were published in the CPTAC endometrial cancer publication.^2^ The composition of patients with different features of interests are shown in Figure 1A. Most of the patients in our cohort have only 1 diagnostic slide (Figure S1B). The independent test set of 137 deidentified FFPE H&E slides from 41 patients at NYU hospitals were retrieved from the archive retrospectively and randomly without any identifiable private information. Due to the nature of endometrial cancer, all the samples in this study presumably came from women. The mixed random data split involved combining samples from TCGA and CPTAC and the dataset were separated into training, validation, and testing sets at per-patient level with a ratio of 8:1:1 for mixed data split trials. The cohort independent data split involved randomly separating TCGA samples with a ratio of 9:1 into training and validation sets and used all CPTAC samples as test set.

### Method details

#### H&E images preparation

Digital histopathologic images were in SVS or SCN format, which are tuples of the same images at multiple different resolutions. Slides from the TCGA cohort were scanned with a maximum resolution of 40x while those from the CPTAC cohort and NYU were at 20x maximum resolution. A Python package, Openslide, was used to maneuver the SVS and SCN files. Due to the extremely large size of these images (Figure S1D), they were cut into small tiles in order to be fed into the training pipeline. Multi-threading was used to accelerate this process. Tiles were cut at 10x, 5x, and 2.5x equivalent resolutions and algorithm was used to exclude tiles with more than 40% pixels of white background and irrelevant contaminants (Figures S1E–S1G). Stain colors of the useful tiles were normalized using the Vahadane’s method during this process.^37^ For each of the tasks, the labels were one-hot encoded at per-tile level. The datasets were separated into training, validation, and testing sets at per-patient level with a ratio of 8:1:1 for mixed data split trials. To take advantage of the Tensorflow API and accelerate the training and testing process, tiles were loaded and saved into a single TFrecords file for each set.

#### Computational method of baseline models

InceptionV1, InceptionV2, InceptionV3, InceptionResnetV1, and InceptionResnetV2 architecture were trained from scratch and used as the baseline models.^31^^,^^36^ InceptionResnets are enhanced architectures of Inceptions with residual connections and a previous study has shown that they are performed generally better than Inceptions in imaging prediction tasks.^31^ The auxiliary classifiers of these architectures were opened. We did not modify any part of the backbone of these architectures. Tiles with 10x resolution were input and we used back-propagation, softmax cross entropy loss weighed by training data composition, and Adam optimization algorithm in the training workflow. Here, each single tile image with a label was considered 1 sample. Batch sizes were set to 64 with an initial learning rate of 0.0001 and a drop-out keep rate of 0.3. The training jobs were run with no fixed epoch number. 100 batches of validation were carried out every 1000 iterations of training and when the training loss achieved a new minimum value after 30000 iterations of training. If the mean of these 100-batch validation loss achieved minimum, the model was saved as the temporary best performing model. The training process stopped when the validation loss did not decrease for at least 10000 iterations. This stopping criterion was only initiated after 100000 iterations of training.

#### Computational method of Panoptes models

We used 4 different Panoptes architectures with and without the integration of patients’ BMI and age in a fourth branch. Panoptes1 has 3 branches based on InceptionResnet1 and Panoptes2 has 3 branches based on InceptionResnet2. The major difference between Panoptes3 and Panoptes1 and between Panoptes4 and Panoptes2 is the additional 1-by-1 convolutional layer between the concatenation of branches and the global average pooling. All of our Panoptes architectures were trained with randomly initialized network parameters with auxiliary classifiers opened on each branch. Unlike the baseline models, tiles of 10x, 5x, and 2.5x resolutions of the same region on the H&E slide with label were paired and considered as 1 sample as only 1 prediction score was associated with a multi-resolution matrix. Batch size was set to 24, which was the largest number that could fit in the memory of our GPUs. Optimization algorithm, weighted loss function, and other hyperparameters were the same as the baselines. In addition, we applied the same validation method to pick the best performing models and kept the same stopping criterion as the baselines.

#### Feature visualization based on tiles

For models with per-patient level AUROC above 0.75 of the test set, we randomly sampled 20000 tiles (tile sets for Panoptes) together with their feature maps before the last fully connected layer in the model, in which each tile or tile set is represented as a 1-dimensional vector. We then used tSNE with initial dimensions of 100 to reduce these 20000 vectors into 2-dimensional space where each point represents a tile or tile set. Generally, points clustered according to their predicted class. By replacing the points on tSNE plots with the original tiles, the features learned by the model for each of the specific class can be observed. We asked experienced pathologists to summarize the typical histological features in each of these clusters.

#### Feature visualization at whole slide level

We built an implementation pipeline that could apply trained models to whole H&E slides and output predictions as heatmaps. The heatmaps could be overlaid on the original slides, which showed the prediction results of different areas. The maximum prediction resolution (each cell of the heatmap) is 299 by 299 pixel at 10x resolution level. Depending on the size of the H&E slides, the time of predicting an intact H&E slides can range from 2 to 40 min. The average speed of prediction with Panoptes models is 22 tile-sets per second, or 1310 tile-sets per minute.

#### Randomization and replication

The digitized H&E slides were randomly split into training, validation, and test sets at 8:1:1 ratio at per-patient level using the python language default randomization algorithm. This ensured that the slides from the same patient were always in the same set, which prevented data leakage issue. The random split was checked for all the prediction tasks to make sure at least 1 positively labeled slide and 1 negatively labeled slide were present in each of the training, validation, and test sets. The same data split was used for all models for the same task to create a fair performance comparison. The overall distribution of positive and negative samples was maintained in the training, validation, and test sets. We repeated the training, validation, and testing for models to test multiple combinations of hyperparameters, including batch size (18, 24, 32, 64), initial learning rate (0.00001, 0.0001, 0.001), and drop-out keep rate (0.3, 0.5, 0.7), and found the best one that achieved optimal results for most tasks mentioned in computational method section. No blinding was involved for this retrospective computational study.

#### Inclusion and exclusion criteria

Only published endometrial tumor H&E slides from TCGA and CPTAC were included. Published normal tissue H&E slides were excluded from this study even if the patient was diagnosis with endometrial cancer. During the tiling and color normalization process, areas of the slide with more than 40% pixels of white background and irrelevant contaminants (RGB values less than 50 or greater than 200 for all three channels) were discarded.

### Quantification and statistical analysis

The performance was evaluated by applying the trained models to the test set. Each of the classification tasks has its own test set, which consists of slides from patients that had not been in the training or validation sets. Evaluation was performed at both per-patient level and per-tile level. Per-patient level metrics were obtained by taking the mean of all tiles’ metrics that belonged to the same patient. For Panoptes models, a 3-multi-resolution-tile matrix is considered as 1 tile for statistical analyses. Receiver Operating Characteristic (ROC) curve, plotting true positive rate against false positive rate, and the area under the ROC curve (AUROC) were the major factors in evaluation. In addition, Precision Recall Curve (PRC), as well as average precision score (AUPR score), were used to determine the trade-off between false negative rate and false positive rate. We also used accuracy with softmax prediction score directly from the models. If the prediction score was greater than 0.5, it was counted as a positively predicted case. 95% Confidence intervals (CI) of AUROC, AUPR, and accuracy were estimated by the bootstrap method. Other statistical metrics, including sensitivity, specificity, precision, recall, F1 score, etc., were also generated and referred to evaluate the predictive models’ performance (Tables S1, S2, S3, and S4). To further validate the effectiveness of the classification models, we did 1-tail Wilcoxon tests between positive and negative tiles in the test sets for each of the tasks as normal distribution could not be assumed and the data was paired from the same population (Figure 2A). In order to compare performance between Panoptes models and the baselines, for each of the tasks with a patient level AUROC score greater than 0.75, we bootstrapped 50 times at an 80% sampling rate at both patient and tile level and calculated the AUROC for each of these sampled sets. The bootstrap random sampling results met the normal distribution assumption for t tests. Therefore, an unpaired 1-tail t test between the AUROC of Panoptes and its corresponding baseline model was performed (Figures 2D, 2E, S3A, and S3B). We performed a similar t test between Panoptes with and without the additional convolutional layer as well as between Panoptes with and without the fourth branch of patients’ BMI and age (Figures S3C–S3F). Statistical analyses and plotting codes were written in R3.6 and Python3.

## Data Availability

Digitized H&E slides from TCGA are publicly available at NCI-GDC Data Portal. Demographic, genomic, and other clinical features associated with these samples are publicly available at cBioPortal and the original TCGA endometrial cancer publication supplements.^1^ Digitized H&E slides from CPTAC are publicly available at The Cancer Imaging Archive (TCIA). Demographic, genomic, and other clinical features of these patients were published in the CPTAC endometrial cancer publication.^2^ Deidentified digitized H&E slides from NYU reported in this paper will be shared by the lead contact upon request. All original code has been deposited at GitHub and is publicly available as of the date of publication. Links are listed in the Key resources table. Any additional information required to reanalyze the data reported in this work paper is available from the Lead Contact upon request.

## Supplemental information

Document S1. Figures S1–S6

Table S1. Statistical summary of mixed random data split models, related to Figures 2 and 5 and Table 1

Table S2. Statistical summary of cohort independent data split models, related to Figure 5

Table S3. Statistical summary of POLE multi-model systems, related to Figure 5 and Table 1

Table S4. Statistical summary of performance on the independent clinical dataset, related to Figure 6

Document S2. Article plus supplemental information
