# Supplementary material for: Predicting endometrial cancer subtypes and molecular features from histopathology images using multi-resolution deep learning models
Source: Cell Rep Med. 2021 Sep 23;2(9):100400. doi: 10.1016/j.xcrm.2021.100400 (PMC8484685; doi:10.1016/j.xcrm.2021.100400)
Supplement: Document S1. Figures S1–S6 [file mmc1.pdf]

Cell Reports Medicine, Volume *2*

Supplemental information

Predicting endometrial cancer subtypes and molecular features from histopathology images using multi-resolution deep learning models

Runyu Hong, Wenke Liu, Deborah DeLair, Narges Razavian, and David Fenyö

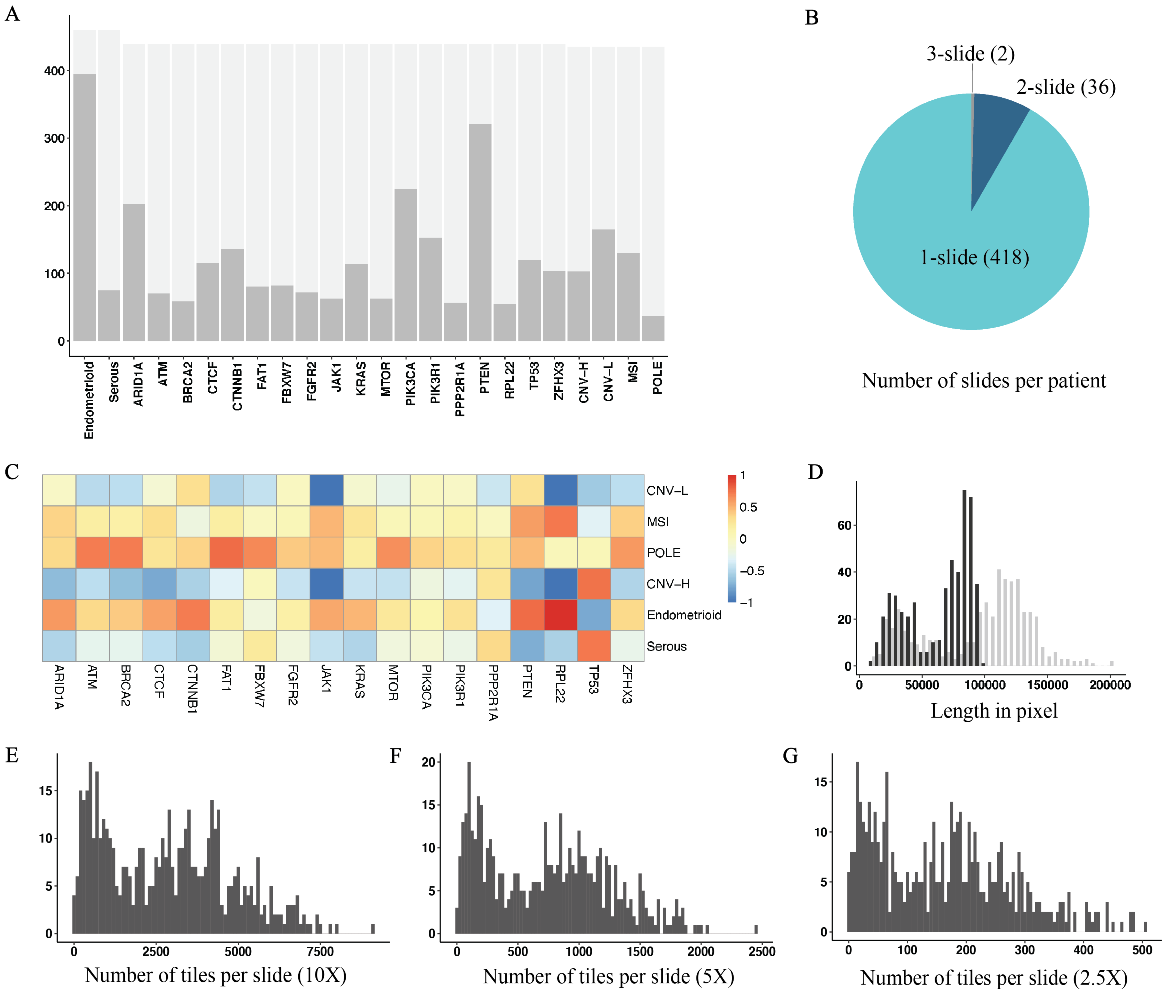

Figure S1. Data summary. **Related to Figure 1.**
(A) Number of patients and composition of true labels in each task. (B) Number of slides per patient in the cohort. (C) Coefficient of colligation between subtypes and mutations. (D) Dimensions of slides in pixel (black: height; grey: width). (E, F, G) Number of tiles per slide at 10X (E), 5X (F), and 2.5X (G) equivalent resolution.

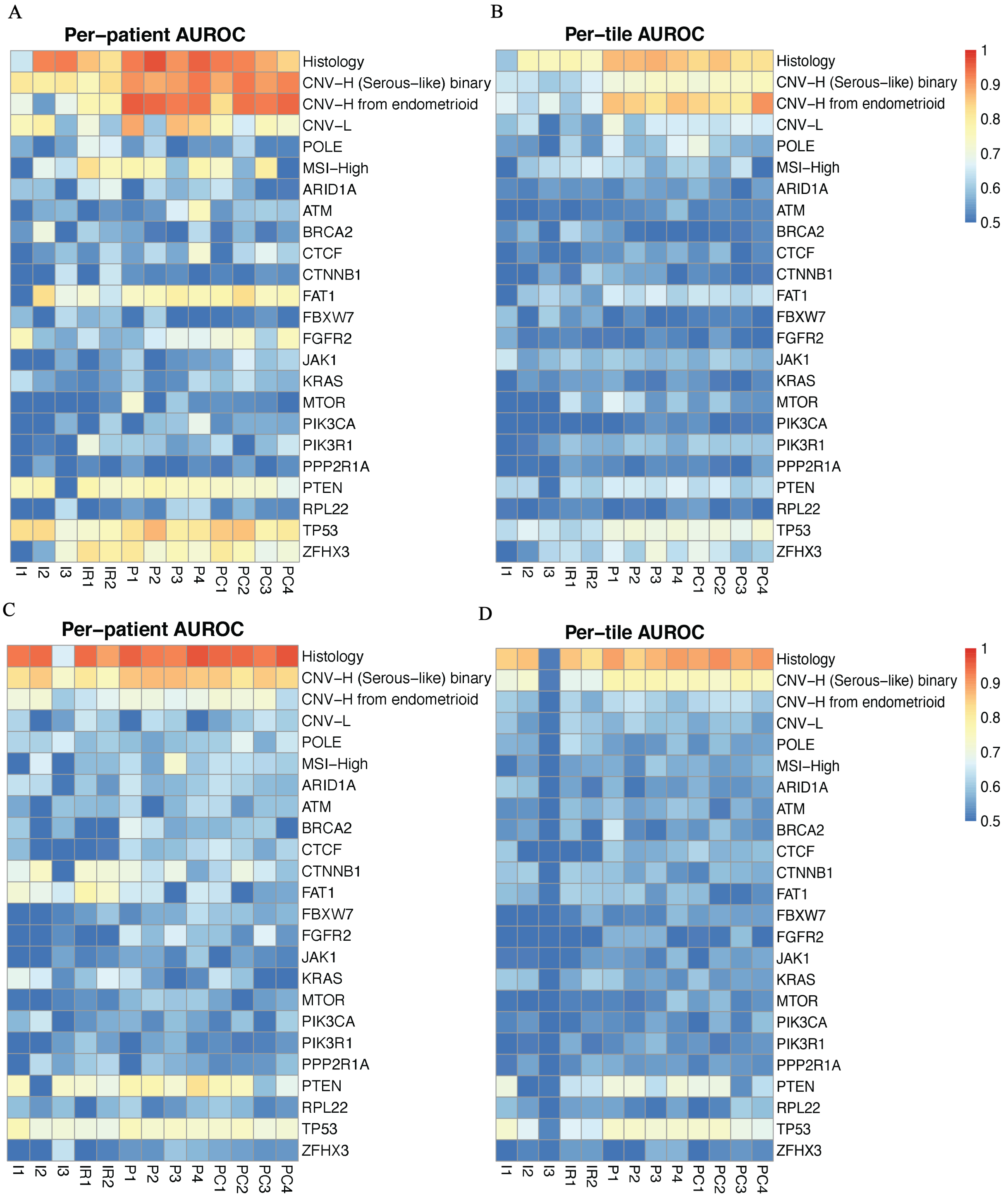

Figure S2. AUROC achieved for Panoptes and baseline models on each prediction task using mixed random data split (A, B) and cohort independent data split (C, D) at per-patient and per-tile level. **Related to Table 1 and Figure 2.** P represents Panoptes, PC represents Panoptes with clinical features, I represents Inception, and IR represents InceptionResnet.

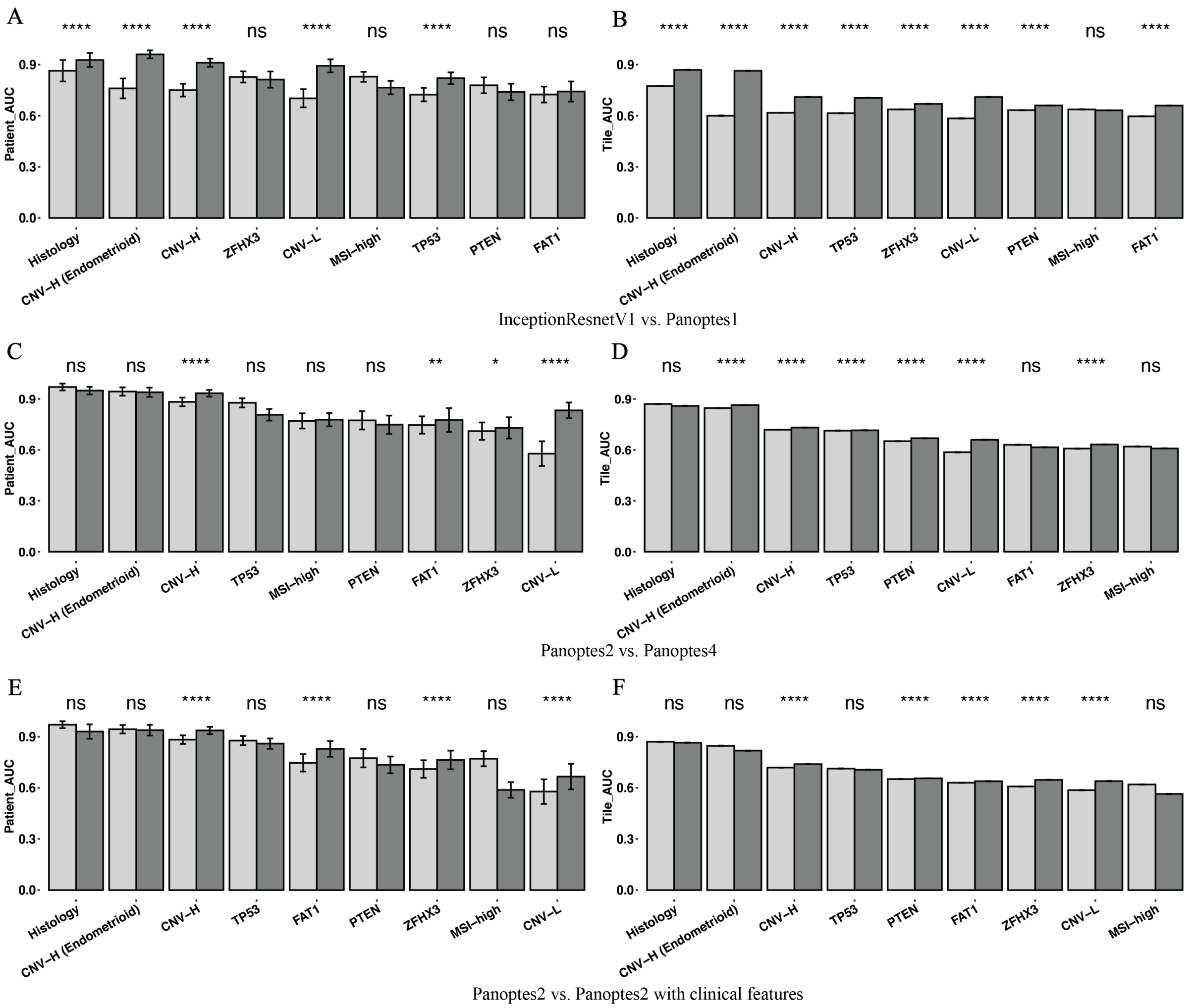

Figure S3. Comparisons of AUROC between architectures on the top eight prediction tasks. **Related to Figure 2.** (A, B) 1-tail t-test of per-patient (A) and per-tile (B) AUROC between InceptionResnetV1 (light) and Panoptes1 (dark) of the top nine tasks. (C, D) 1-tail t-test of per-patient (C) and per-tile (D) AUROC of Panoptes2 (light) and Panoptes4 (dark) of top nine tasks. (E, F) Bootstrapped per-patient (E) and per-tile (F) 1-tail t-test of AUROC of Panoptes2 (light) and Panoptes2 with clinical features (dark) of top nine tasks.

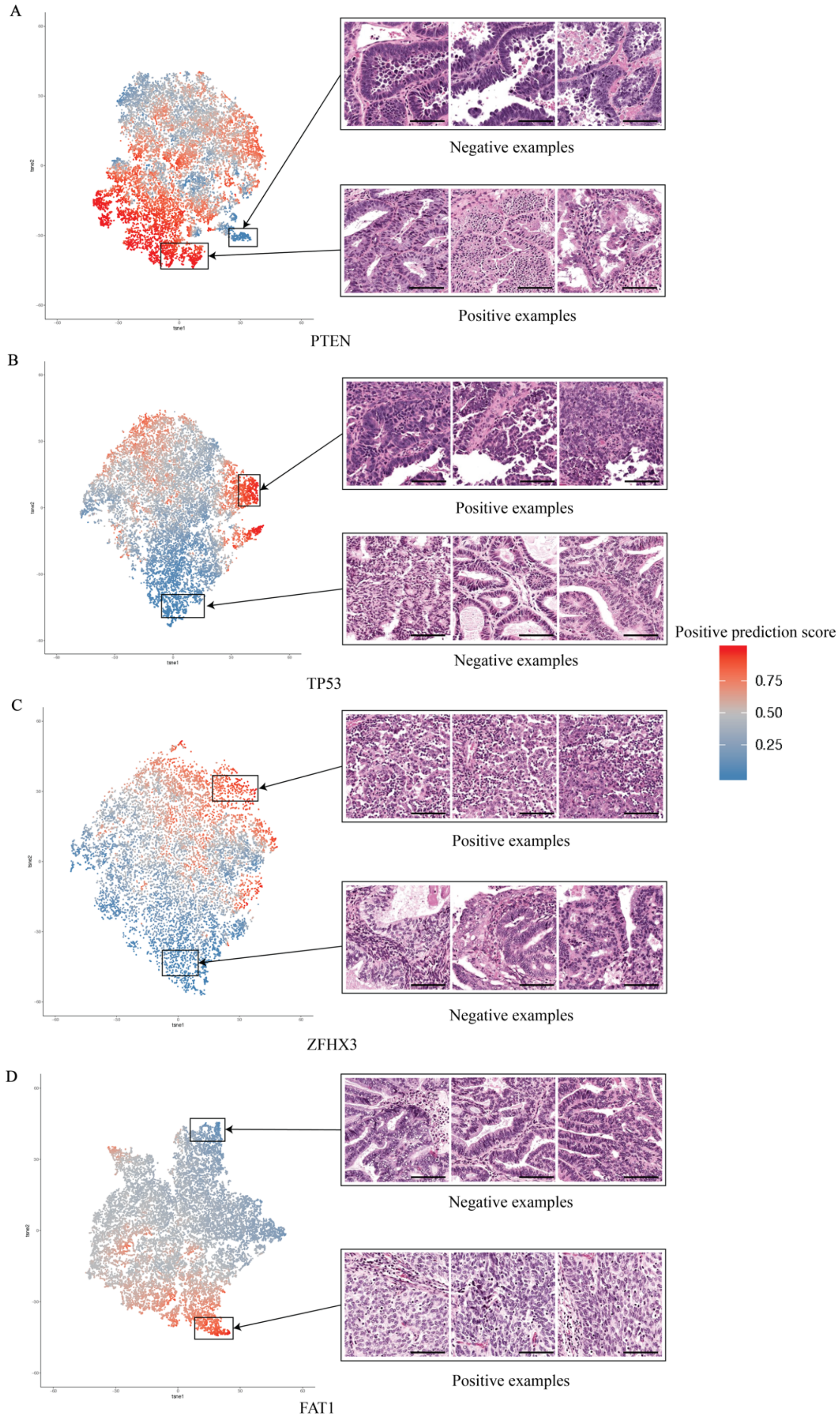

Figure S4. Extraction and visualization of features learned by the models with tSNE. **Related to Figure 3.** Each point represents a tile and is colored according to its corresponding positive prediction score. Scale bars represent 100µm. (A) *PTEN* from a Panoptes2 model. (B) *TP53* from a Panoptes2 model. (C) *ZFHX3* from a Panoptes1 model. (D) *FAT1* from a Panoptes2 with clinical features model.

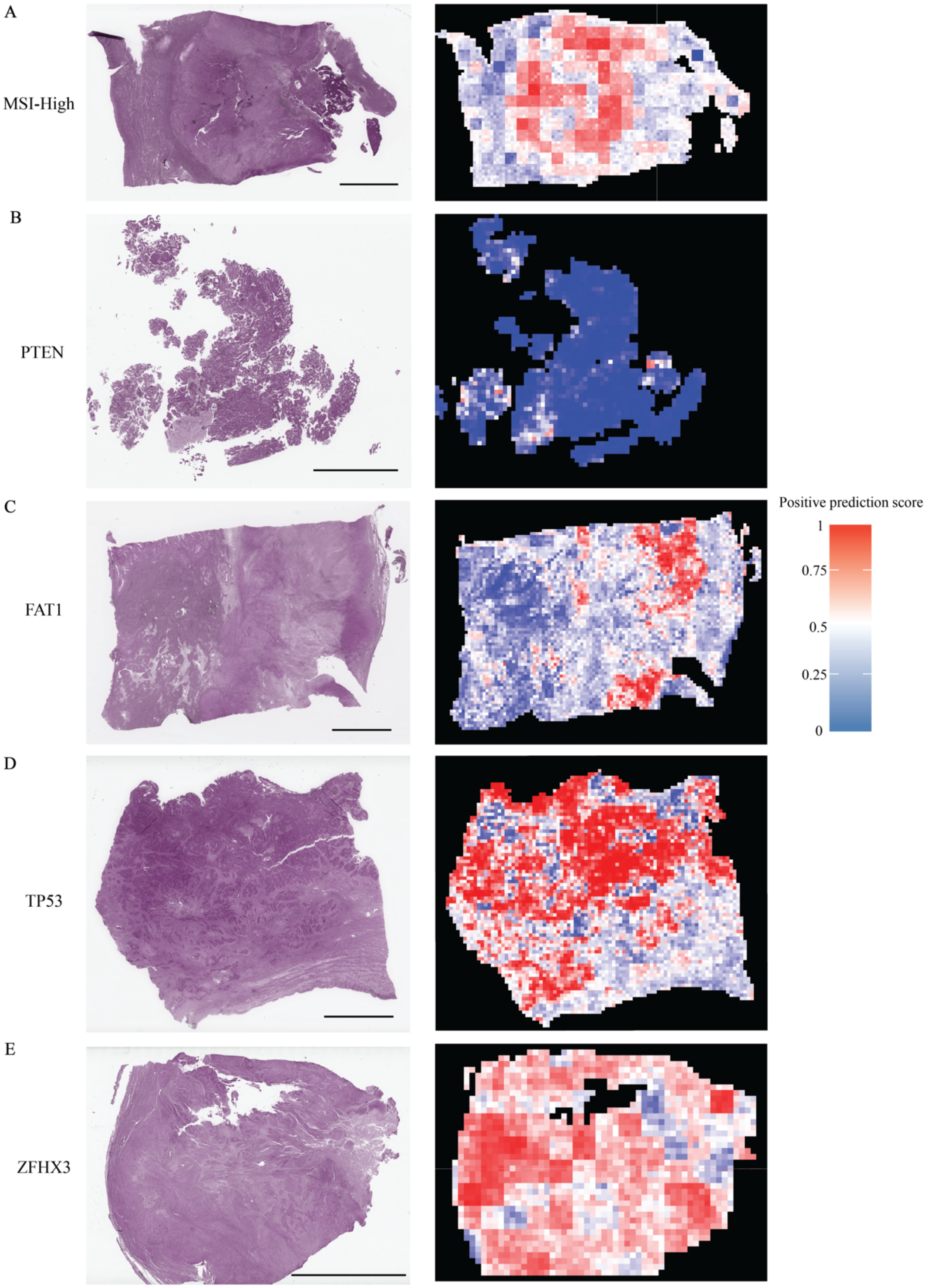

Figure S5. Whole slide predictions with color representing positive prediction scores. Scale bars represent 5000µm. **Related to Figure 4.**

(A) Slide from an MSI-High (positive) patient using a Panoptes1 model. (B) Slide from a *PTEN* wild-type (negative) patient using a Panoptes2 model. (C) Slide from a *FAT1* mutated (positive) patient using a Panoptes3 model. (D) Slide from a *TP53* mutated (positive) patient using a Panoptes2 model. (E) Slide from a *ZFHX3* mutated (positive) patient using a Panoptes1 model.

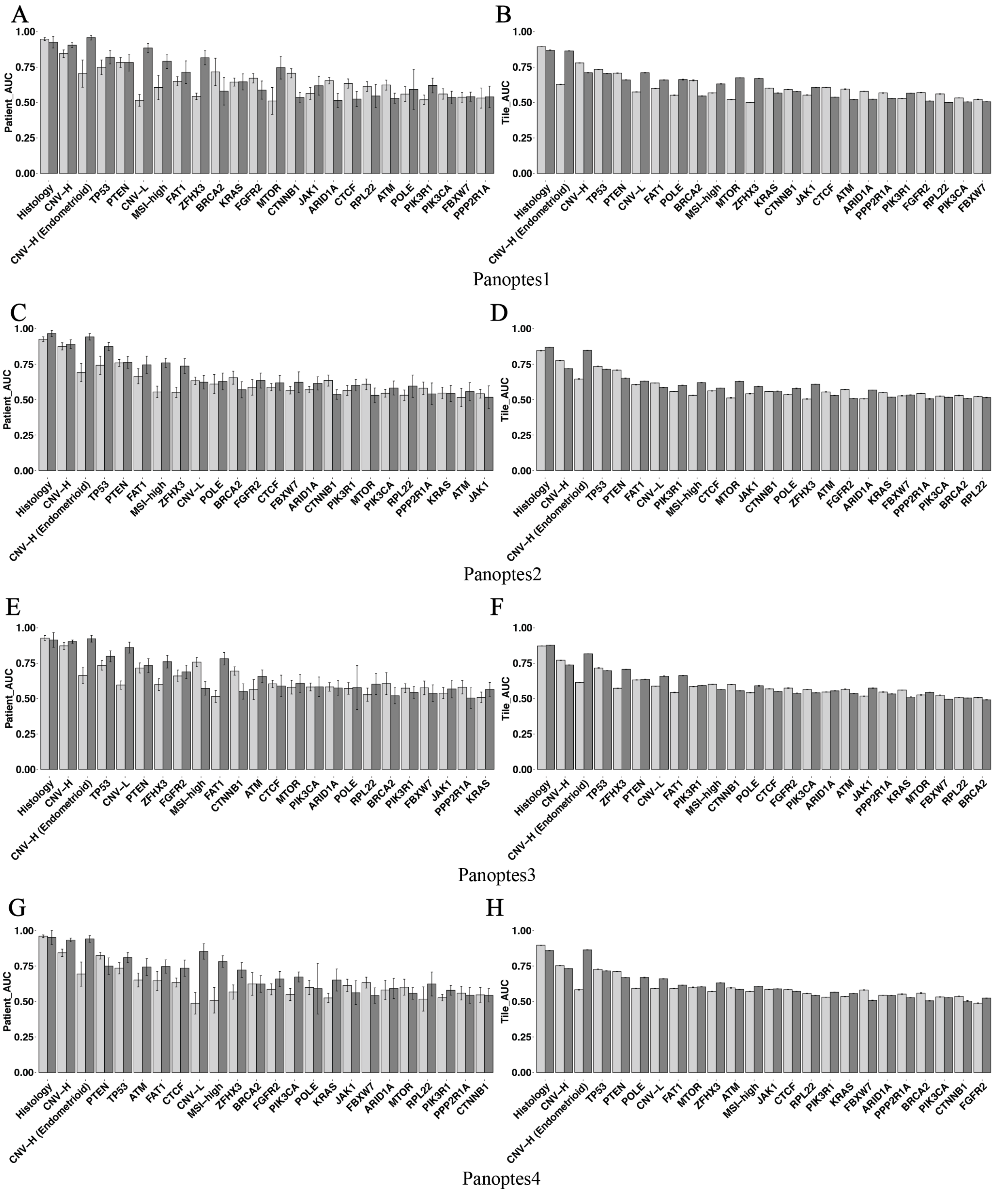

Figure S6. Comparisons of AUROC between the Panoptes models in mixed random split trials and cohort independent split trials. **Related to Figure 5.**

Per-patient and per-tile level AUROC of Panoptes1 (A, B), Panoptes2 (C, D), Panoptes3 (E, F), and Panoptes4 (G, H) models in each task with mixed random data split (dark) and the cohort independent data split (light).
